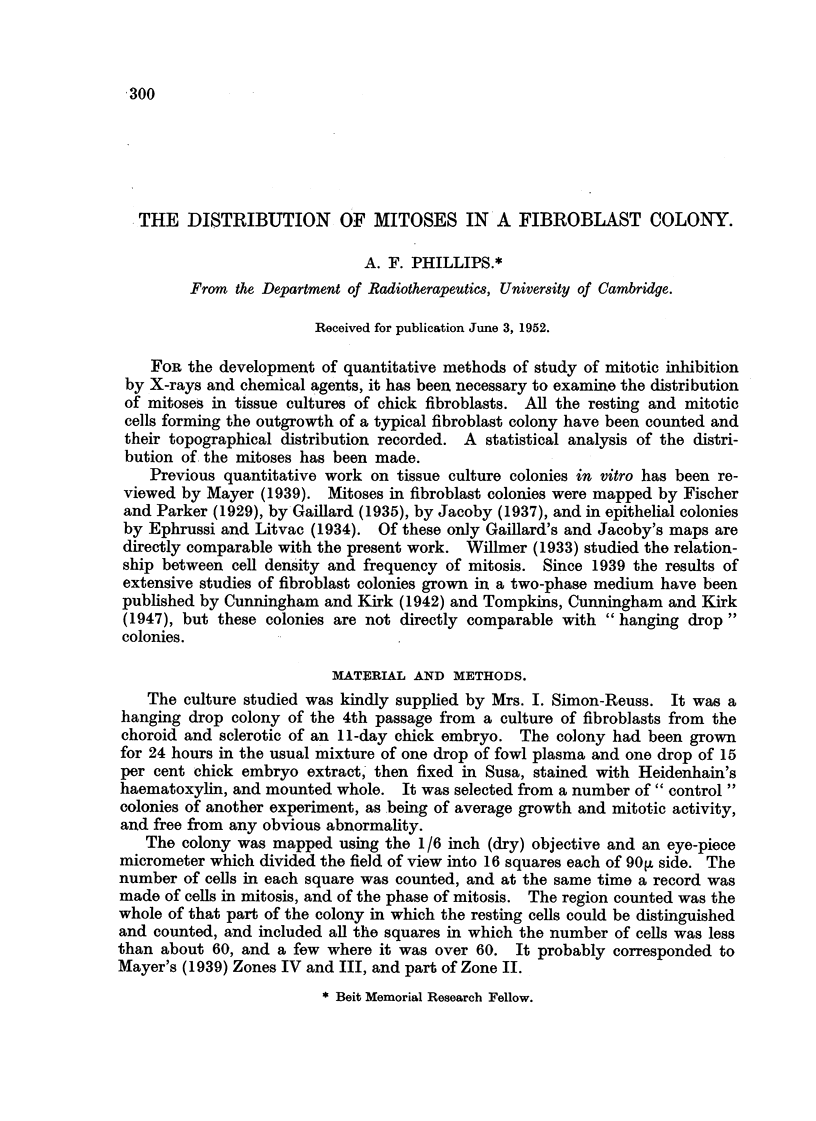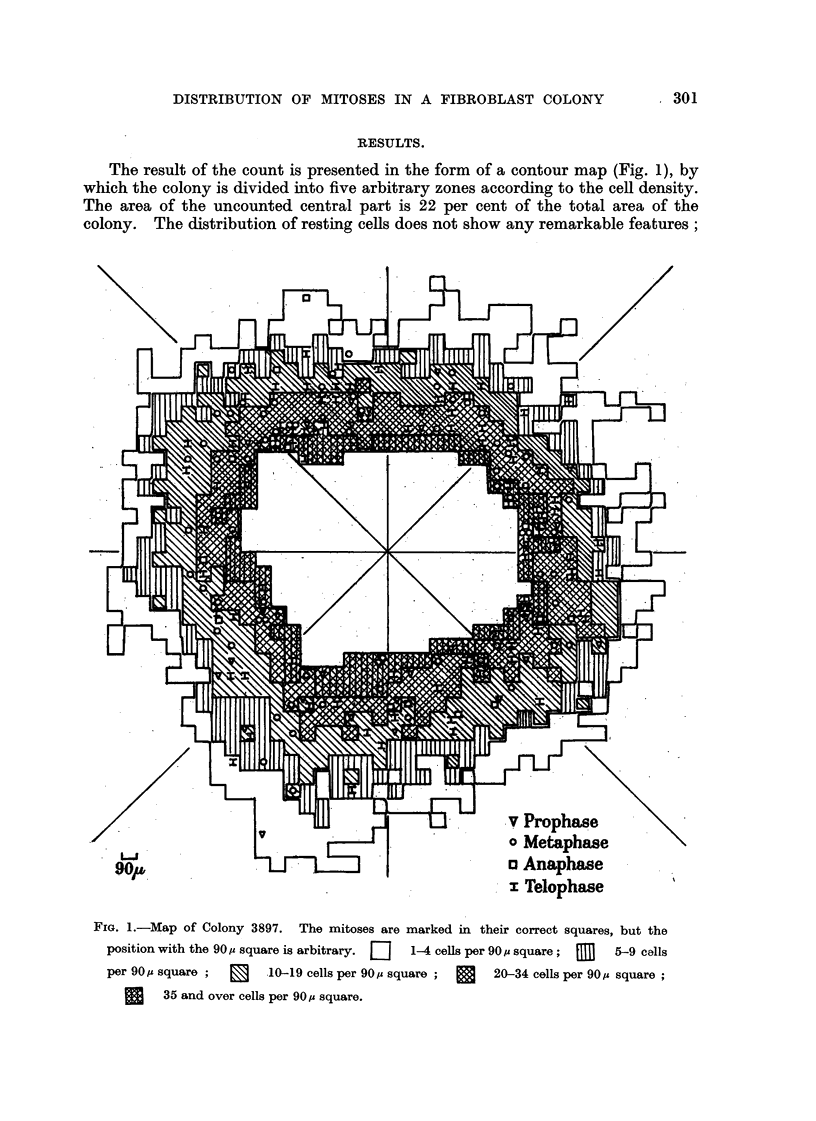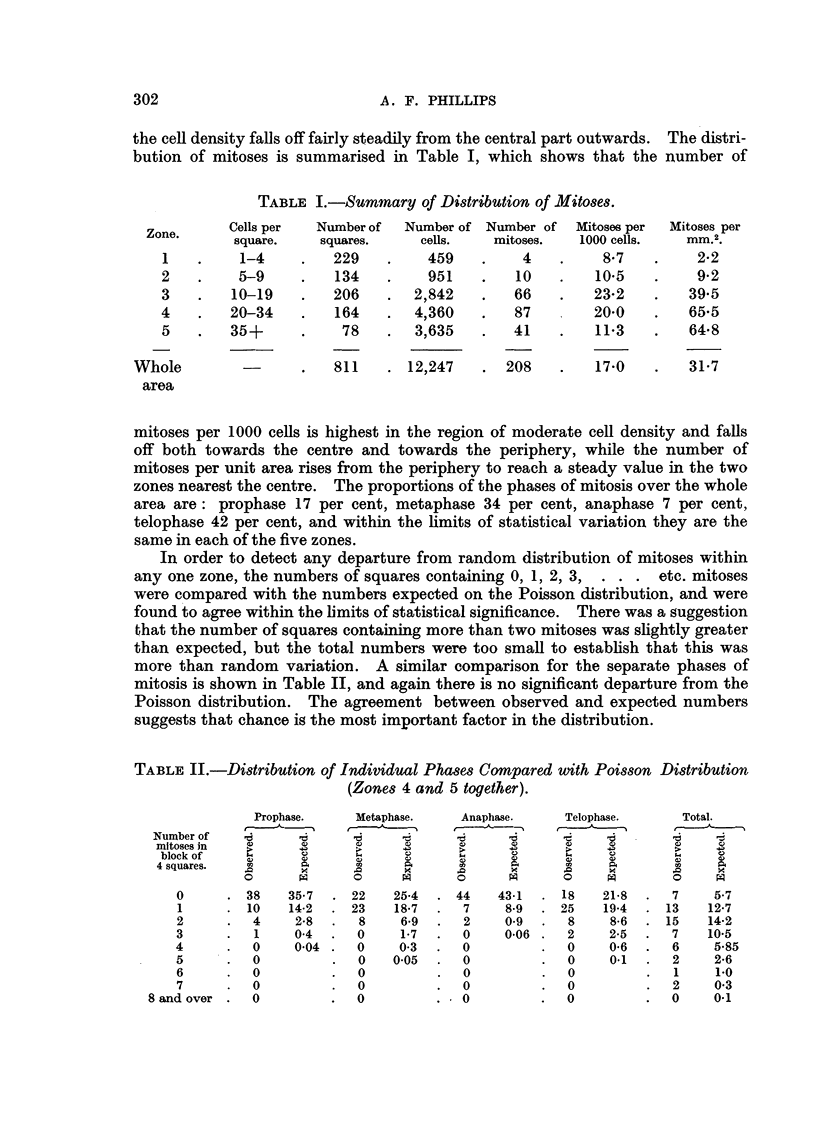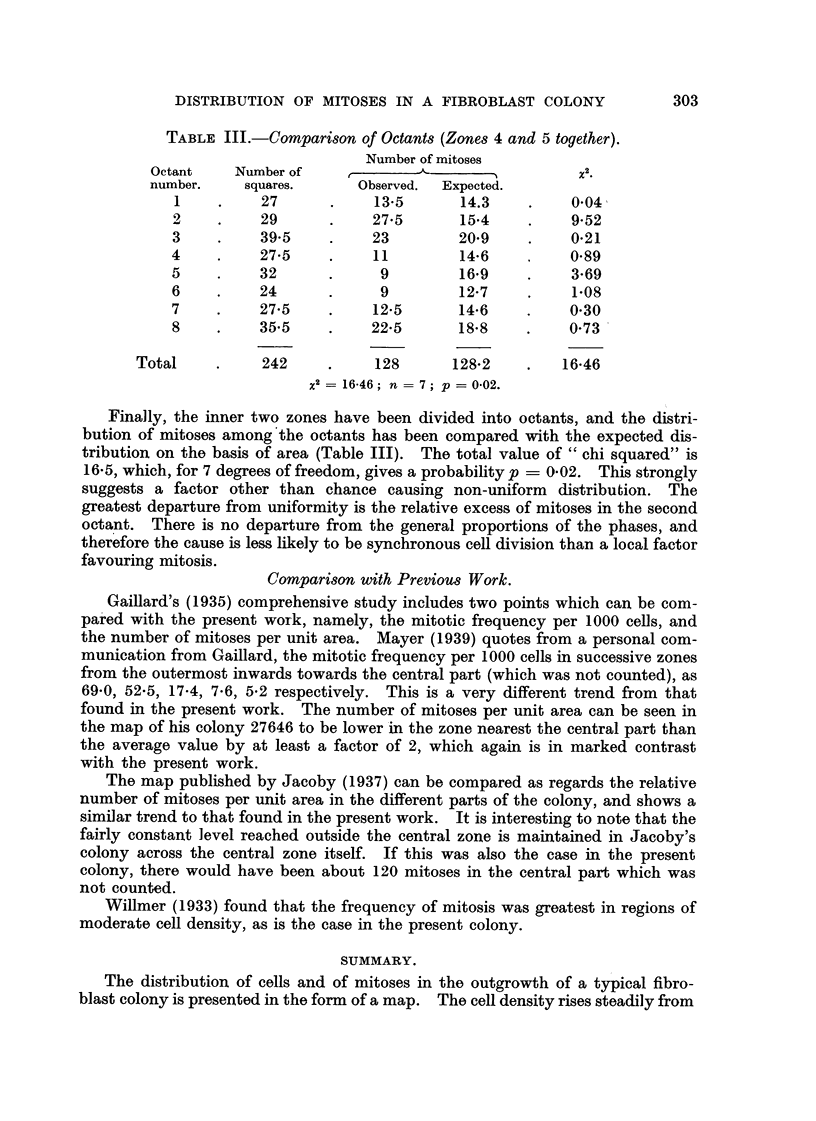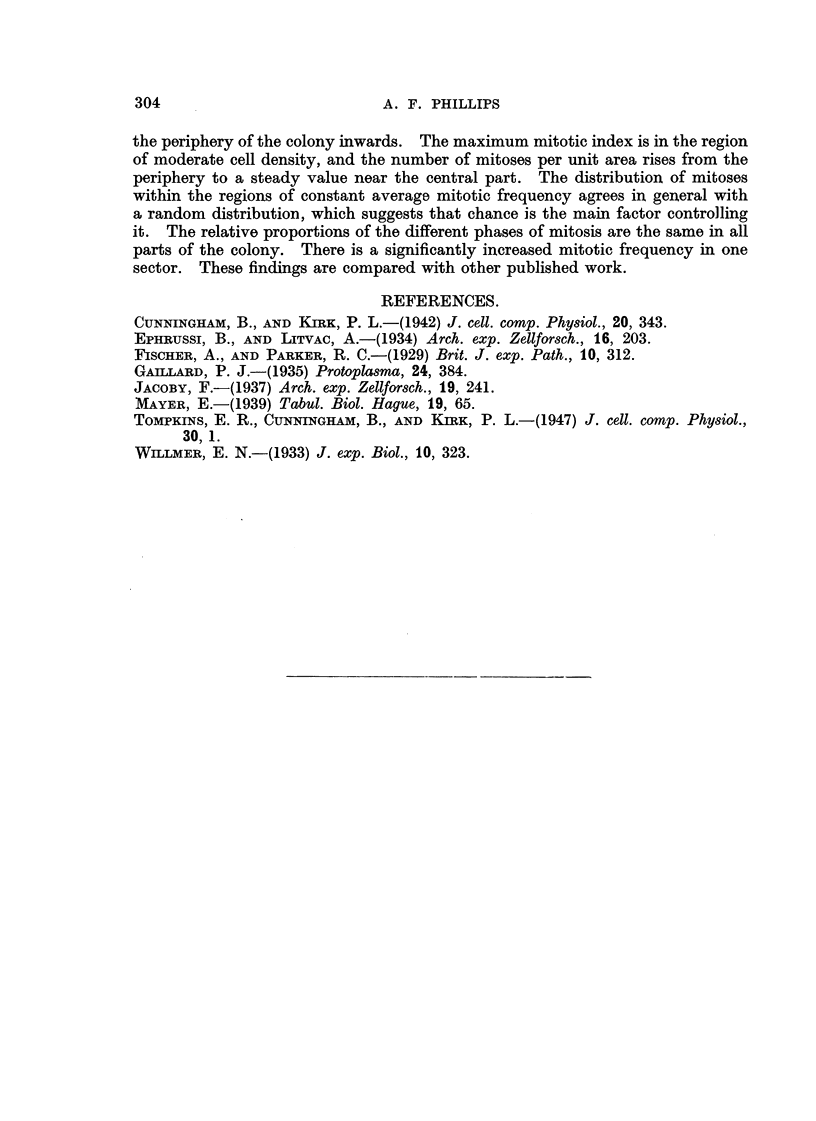# The Distribution of Mitoses in a Fibroblast Colony

**DOI:** 10.1038/bjc.1952.34

**Published:** 1952-09

**Authors:** A. F. Phillips


### THE DISTRIBUTION OF MITOSES IN A FIBROBLAST COLONY.

A. F. PHILLIPS.*

*From the Department of Radiotherapeutics, University of Cambridge.*

Received for publication June 3, 1952.

FOR the development of quantitative methods of study of mitotic inhibition by X-rays and chemical agents, it has been necessary to examine the distribution of mitoses in tissue cultures of chick fibroblasts. All the resting and mitotic cells forming the outgrowth of a typical fibroblast colony have been counted and their topographical distribution recorded. A statistical analysis of the distribution of the mitoses has been made.

Previous quantitative work on tissue culture colonies *in vitro* has been reviewed by Mayer (1939). Mitoses in fibroblast colonies were mapped by Fischer and Parker (1929), by Gaillard (1935), by Jacoby (1937), and in epithelial colonies by Ephrussi and Litvac (1934). Of these only Gaillard's and Jacoby's maps are directly comparable with the present work. Willmer (1933) studied the relationship between cell density and frequency of mitosis. Since 1939 the results of extensive studies of fibroblast colonies grown in a two-phase medium have been published by Cunningham and Kirk (1942) and Tompkins, Cunningham and Kirk (1947), but these colonies are not directly comparable with " hanging drop " colonies.

### MATERIAL AND METHODS.

The culture studied was kindly supplied by Mrs. I. Simon-Reuss. It was a hanging drop colony of the 4th passage from a culture of fibroblasts from the choroid and sclerotic of an 11-day chick embryo. The colony had been grown for 24 hours in the usual mixture of one drop of fowl plasma and one drop of 15 per cent chick embryo extract, then fixed in Susa, stained with Heidenhain's haematoxylin, and mounted whole. It was selected from a number of " control " colonies of another experiment, as being of average growth and mitotic activity, and free from any obvious abnormality.

The colony was mapped using the 1/6 inch (dry) objective and an eye-piece micrometer which divided the field of view into 16 squares each of $90\mu$ side. The number of cells in each square was counted, and at the same time a record was made of cells in mitosis, and of the phase of mitosis. The region counted was the whole of that part of the colony in which the resting cells could be distinguished and counted, and included all the squares in which the number of cells was less than about 60, and a few where it was over 60. It probably corresponded to Mayer's (1939) Zones IV and III, and part of Zone II.

* Beit Memorial Research Fellow.

### RESULTS.

The result of the count is presented in the form of a contour map (Fig. 1), by which the colony is divided into five arbitrary zones according to the cell density. The area of the uncounted central part is 22 per cent of the total area of the colony. The distribution of resting cells does not show any remarkable features ;

FIG. 1.—Map of Colony 3897. The mitoses are marked in their correct squares, but the position with the 90μ square is arbitrary. □ 1–4 cells per 90μ square ; ▥ 5–9 cells per 90μ square ; ▧ 10–19 cells per 90μ square ; ▩ 20–34 cells per 90μ square ; ▦ 35 and over cells per 90μ square.

the cell density falls off fairly steadily from the central part outwards. The distribution of mitoses is summarised in Table I, which shows that the number of

TABLE I.—*Summary of Distribution of Mitoses.*

| Zone. | Cells per square. | Number of squares. | Number of cells. | Number of mitoses. | Mitoses per 1000 cells. | Mitoses per mm.$^2$. |
|---|---|---|---|---|---|---|
| 1 | 1–4 | 229 | 459 | 4 | 8·7 | 2·2 |
| 2 | 5–9 | 134 | 951 | 10 | 10·5 | 9·2 |
| 3 | 10–19 | 206 | 2,842 | 66 | 23·2 | 39·5 |
| 4 | 20–34 | 164 | 4,360 | 87 | 20·0 | 65·5 |
| 5 | 35+ | 78 | 3,635 | 41 | 11·3 | 64·8 |
| Whole area | — | 811 | 12,247 | 208 | 17·0 | 31·7 |

mitoses per 1000 cells is highest in the region of moderate cell density and falls off both towards the centre and towards the periphery, while the number of mitoses per unit area rises from the periphery to reach a steady value in the two zones nearest the centre. The proportions of the phases of mitosis over the whole area are: prophase 17 per cent, metaphase 34 per cent, anaphase 7 per cent, telophase 42 per cent, and within the limits of statistical variation they are the same in each of the five zones.

In order to detect any departure from random distribution of mitoses within any one zone, the numbers of squares containing 0, 1, 2, 3, . . . etc. mitoses were compared with the numbers expected on the Poisson distribution, and were found to agree within the limits of statistical significance. There was a suggestion that the number of squares containing more than two mitoses was slightly greater than expected, but the total numbers were too small to establish that this was more than random variation. A similar comparison for the separate phases of mitosis is shown in Table II, and again there is no significant departure from the Poisson distribution. The agreement between observed and expected numbers suggests that chance is the most important factor in the distribution.

TABLE II.—*Distribution of Individual Phases Compared with Poisson Distribution (Zones 4 and 5 together).*

| Number of mitoses in block of 4 squares. | Prophase. | | Metaphase. | | Anaphase. | | Telophase. | | Total. | |
|---|---|---|---|---|---|---|---|---|---|---|
| | Observed. | Expected. | Observed. | Expected. | Observed. | Expected. | Observed. | Expected. | Observed. | Expected. |
| 0 | 38 | 35·7 | 22 | 25·4 | 44 | 43·1 | 18 | 21·8 | 7 | 5·7 |
| 1 | 10 | 14·2 | 23 | 18·7 | 7 | 8·9 | 25 | 19·4 | 13 | 12·7 |
| 2 | 4 | 2·8 | 8 | 6·9 | 2 | 0·9 | 8 | 8·6 | 15 | 14·2 |
| 3 | 1 | 0·4 | 0 | 1·7 | 0 | 0·06 | 2 | 2·5 | 7 | 10·5 |
| 4 | 0 | 0·04 | 0 | 0·3 | 0 | | 0 | 0·6 | 6 | 5·85 |
| 5 | 0 | | 0 | 0·05 | 0 | | 0 | 0·1 | 2 | 2·6 |
| 6 | 0 | | 0 | | 0 | | 0 | | 1 | 1·0 |
| 7 | 0 | | 0 | | 0 | | 0 | | 2 | 0·3 |
| 8 and over | 0 | | 0 | | 0 | | 0 | | 0 | 0·1 |

TABLE III.—*Comparison of Octants (Zones 4 and 5 together).*

| Octant number. | Number of squares. | Number of mitoses Observed. | Number of mitoses Expected. | $\chi^2$. |
|---|---|---|---|---|
| 1 | 27 | 13·5 | 14.3 | 0·04 |
| 2 | 29 | 27·5 | 15·4 | 9·52 |
| 3 | 39·5 | 23 | 20·9 | 0·21 |
| 4 | 27·5 | 11 | 14·6 | 0·89 |
| 5 | 32 | 9 | 16·9 | 3·69 |
| 6 | 24 | 9 | 12·7 | 1·08 |
| 7 | 27·5 | 12·5 | 14·6 | 0·30 |
| 8 | 35·5 | 22·5 | 18·8 | 0·73 |
| Total | 242 | 128 | 128·2 | 16·46 |

$\chi^2 = 16{\cdot}46$ ; $n = 7$ ; $p = 0{\cdot}02$.

Finally, the inner two zones have been divided into octants, and the distribution of mitoses among the octants has been compared with the expected distribution on the basis of area (Table III). The total value of "chi squared" is 16·5, which, for 7 degrees of freedom, gives a probability $p = 0{\cdot}02$. This strongly suggests a factor other than chance causing non-uniform distribution. The greatest departure from uniformity is the relative excess of mitoses in the second octant. There is no departure from the general proportions of the phases, and therefore the cause is less likely to be synchronous cell division than a local factor favouring mitosis.

### *Comparison with Previous Work.*

Gaillard's (1935) comprehensive study includes two points which can be compared with the present work, namely, the mitotic frequency per 1000 cells, and the number of mitoses per unit area. Mayer (1939) quotes from a personal communication from Gaillard, the mitotic frequency per 1000 cells in successive zones from the outermost inwards towards the central part (which was not counted), as 69·0, 52·5, 17·4, 7·6, 5·2 respectively. This is a very different trend from that found in the present work. The number of mitoses per unit area can be seen in the map of his colony 27646 to be lower in the zone nearest the central part than the average value by at least a factor of 2, which again is in marked contrast with the present work.

The map published by Jacoby (1937) can be compared as regards the relative number of mitoses per unit area in the different parts of the colony, and shows a similar trend to that found in the present work. It is interesting to note that the fairly constant level reached outside the central zone is maintained in Jacoby's colony across the central zone itself. If this was also the case in the present colony, there would have been about 120 mitoses in the central part which was not counted.

Willmer (1933) found that the frequency of mitosis was greatest in regions of moderate cell density, as is the case in the present colony.

### SUMMARY.

The distribution of cells and of mitoses in the outgrowth of a typical fibroblast colony is presented in the form of a map. The cell density rises steadily from the periphery of the colony inwards. The maximum mitotic index is in the region of moderate cell density, and the number of mitoses per unit area rises from the periphery to a steady value near the central part. The distribution of mitoses within the regions of constant average mitotic frequency agrees in general with a random distribution, which suggests that chance is the main factor controlling it. The relative proportions of the different phases of mitosis are the same in all parts of the colony. There is a significantly increased mitotic frequency in one sector. These findings are compared with other published work.

### REFERENCES.

Cunningham, B., and Kirk, P. L.—(1942) *J. cell. comp. Physiol.*, **20**, 343.

Ephrussi, B., and Litvac, A.—(1934) *Arch. exp. Zellforsch.*, **16**, 203.

Fischer, A., and Parker, R. C.—(1929) *Brit. J. exp. Path.*, **10**, 312.

Gaillard, P. J.—(1935) *Protoplasma*, **24**, 384.

Jacoby, F.—(1937) *Arch. exp. Zellforsch.*, **19**, 241.

Mayer, E.—(1939) *Tabul. Biol. Hague*, **19**, 65.

Tompkins, E. R., Cunningham, B., and Kirk, P. L.—(1947) *J. cell. comp. Physiol.*, **30**, 1.

Willmer, E. N.—(1933) *J. exp. Biol.*, **10**, 323.